# Supplementary figures and images for: Molecular epidemiological characteristics of dengue virus carried by 34 patients in Guangzhou in 2018
Source: PLoS One. 2019 Nov 14;14(11):e0224676. doi: 10.1371/journal.pone.0224676 (PMC6855448; doi:10.1371/journal.pone.0224676)

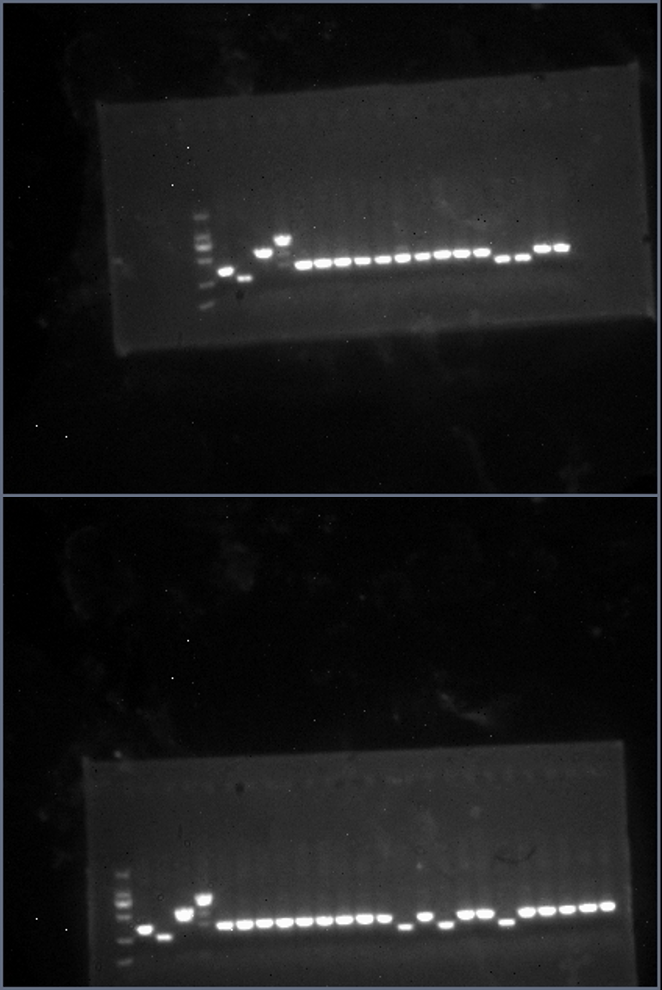

Supplement: S1 Fig — (TIF) [file pone.0224676.s001.tif]
